# The Supportive and Palliative Radiation Oncology Service: A Dedicated Model for Palliative Radiation Oncology Care

**DOI:** 10.6004/jadpro.2015.6.2.5

**Published:** 2015-03-01

**Authors:** Daniel Gorman, Tracy Balboni, Allison Taylor, Monica Krishnan

**Affiliations:** 1 Dana-Farber Cancer Institute; 2 Brigham and Women’s Hospital, Boston, Massachusetts

Radiation therapy (RT) has been an established modality for treating various  types of cancers since the early 1900s. It became more broadly utilized in cancer therapy beginning in the 1960s, with greater accessibility to radioactive sources such as cobalt-60 and the emergence of the linear accelerator. The linear accelerator is a piece of equipment that instead of requiring a radioactive source, uses electricity to generate high-energy photons (x-rays) or charged particles (i.e., electrons; Bernier, Hall, & Giaccia, 2004; Connell & Hellman, 2009; Mitin, 2013). Over the ensuing decades, the specialty of radiation oncology and its technologies have continued to evolve and now have a central role in treating many types of cancers, both as an independent modality and in combination with chemotherapy (Bernier, Hall, & Giaccia, 2004; Connell & Hellman, 2009; Mitin, 2013).

When RT is delivered, it is most often delivered as high-energy photons, which can deeply penetrate tissue, or electrons, which deliver superficial radiation therapy generated by a linear accelerator. Though less commonly used, brachytherapy (placement of radioactive sources into the tumor) is also an important modality for treating tumors. In whatever form, RT requires a *simulation* (a method of reproducibly setting up the patient for each treatment, often with immobilization devices and freckle-sized tattoos as setup points), followed by a CT scan of the region of interest.

Next, radiation planning, in which the tumor target is treated while minimizing the dose to normal structures, is performed. *Conformal planning* is the use of computer modeling of radiation-beam arrangements to optimize targeting of the tumor while sparing normal tissues. *Intensity-modulated radiation therapy* (IMRT) is a type of conformal therapy that allows thin radiation beams of varying intensity to generate high conformality of the dose to the target. *Stereotactic radiosurgery* (SRS) is high dose-per-treatment RT delivered in a single treatment (or up to five treatments) using high-precision radiation beams (Bernier, Hall, & Giaccia, 2004; Connell & Hellman, 2009; Mitin, 2013).

## PALLIATIVE RADIATION THERAPY

Although radiation is commonly used to treat patients with curative intent, approximately 40% of patients undergoing RT receive treatment with palliative intent (Tseng et al., 2014; van Oorschot, Rades, Schulze Beckmann, & Feyer, 2011). Palliative RT’s applications to provide symptomatic relief and to halt disease progression in the context of incurable disease are numerous (Table), such as palliation of bone and brain metastases. Additionally, RT has an important role in many oncologic emergencies, including spinal cord compression, superior vena cava syndrome, and tumor-related hemoptysis or bronchial obstruction.

**Table 1:**
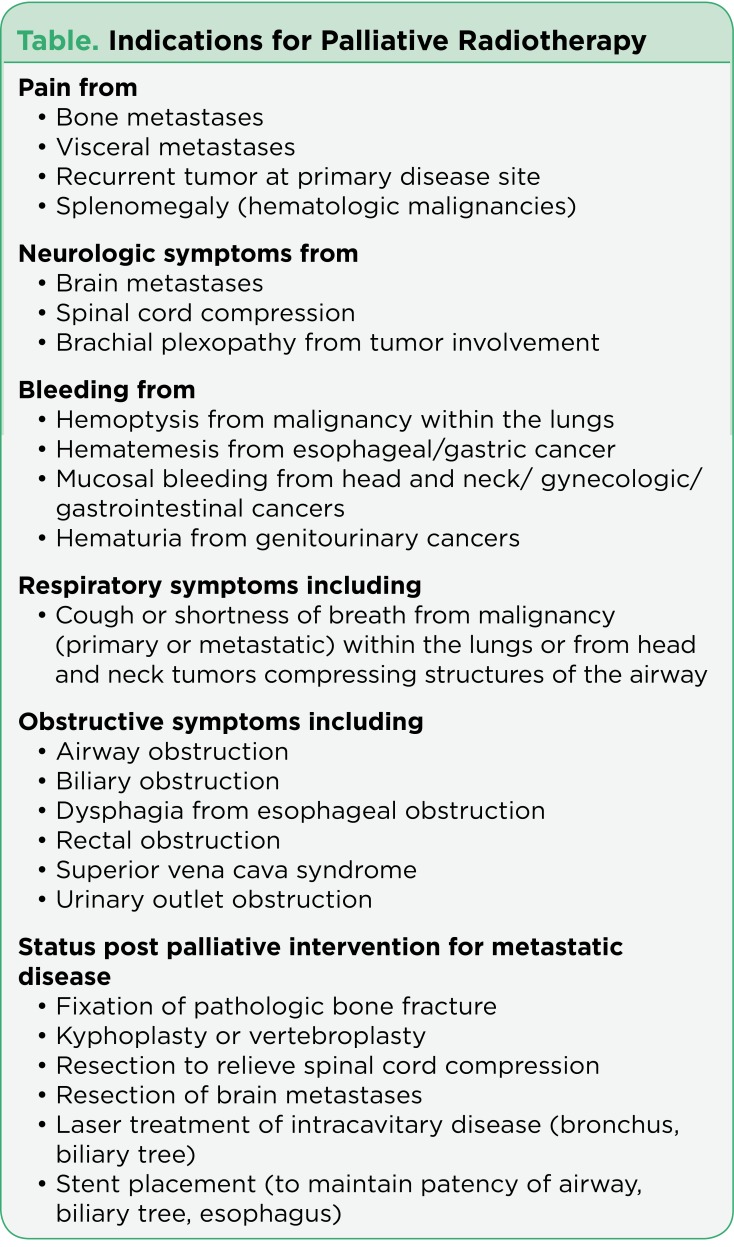
Indications for Palliative Radiotherapy

Patients presenting to radiation oncology departments for consideration of palliative RT have unique and complex issues, which frequently require urgent management. These issues include symptom management, coordination of care with other specialties (e.g., medical and surgical oncology, palliative medicine), psychosocial issues, and patient/family communication. In light of the frequency and complexity of this population of patients seen in radiation oncology, together with national recommendations to establish integrated models of palliative care within oncology (Peppercorn et al., 2011), there is a growing recognition of palliative radiation oncology as a specialty within radiation oncology.

In most cancer institutions, palliative RT consultations are handled by radiation oncologists within their respective disease groups or by "on-call" teams. However, the departmental systems of care for these patients are frequently not tailored to their complex and urgent needs; for example, at busy academic centers these patients are often added on to the radiation oncologist’s otherwise full schedule.

Furthermore, radiation oncology providers typically have no specialized training in palliative care, as most training programs do not have focused training in palliative oncology care. Given the complexity of this patient population, the development of dedicated programs to meet the palliative oncology care needs of these patients is required, particularly at academic cancer centers, where the volume of these patients is high and training and research to advance care can concurrently be implemented. Dedicated palliative radiation oncology programs already exist in Canada, including longstanding programs at two hospitals in Toronto (Chow & Holden, 2011).

## MODEL OF PALLIATIVE RADIATION ONCOLOGY

Following these models of dedicated palliative radiation oncology care, the Dana-Farber/Brigham and Women’s Cancer Center (DFBWCC) Department of Radiation Oncology, together with the support of the DFBWCC Department of Psychosocial Oncology and Palliative Medicine, aimed to build a dedicated model of palliative radiation oncology care, the first in the United States. The DFBWCC Supportive and Palliative Radiation Oncology (SPRO) Service, initiated in July 2011, was developed to provide and advance excellence in radiation oncology care to patients facing advanced cancers.

**Leadership and Staff**

The SPRO service has a shared physician/nurse practitioner leadership model. The physician clinical director is Board-certified in both radiation oncology and palliative medicine. The assistant director is a nurse practitioner with a master’s degree in health-care administration, advanced training in palliative care, as well as medical oncology. Within the first year of the program, a part-time nurse practitioner, with extensive experience in radiation oncology and as a hospice nurse, was added to meet the demands of the service.

The SPRO team consists of weekly rotating attending and resident radiation oncology physicians and a dedicated nurse practitioner. The service also has a dedicated program administrator who is critical in helping to coordinate consults, obtain records, interface with outside referring physicians, and communicate directly with patients and families. The registered nurse staff rotates based on the attending covering the SPRO service on a weekly basis. Nurses work closely with the nurse practitioner and physician staff in managing patients, from treatment planning through completion of their treatment.

**Clinical Activities**

The SPRO service provides a dedicated clinical team available 24 hours a day, 7 days a week to care for advanced cancer patients’ complex and urgent clinical care needs. Communication with the SPRO service is via a single call-in location—a dedicated pager—covered by the nurse practitioner during the day and the resident after hours and weekends. The nurse practitioner or resident, in consultation with the team, performs patient triage and communicates with the referring clinician; with administrative assistance of the SPRO administrator, the nurse practitioner or resident arranges for the patient to be seen in consultation in a time frame appropriate to the clinical presentation. This can often vary from immediate evaluation to evaluation within a few days.

The SPRO service performs daily rounds on all active patients, which is often followed by walking rounds. Frequent smaller "huddles" occur throughout the day to maintain communication among the team. Lastly, afternoon rounds occur to wrap up the day’s activities and to prepare for the next day.

The key clinical activities of the service include three components. First, there is a comprehensive evaluation of the patient, including a review of the pertinent imaging. This often includes a more comprehensive evaluation of both physical and psychosocial issues, which are addressed directly where appropriate or through arranging for the involvement of other care providers, such as social work or palliative medicine. Second, there is consideration of the role of radiotherapy and other potential palliative modalities, which often requires communication between various care providers. Third, there is communication with patients and families regarding radiotherapy recommendations and other care communications, such as pharmacologic symptom management, goals of care discussions, family meetings, and collaborative dialogue with the patient’s care team.

In recognition of the frequency of complex spine cases requiring care coordination among spine surgery, interventional radiology, and radiation oncology, the DFBWCC Multidisciplinary Spine Oncology Conference was established in 2012. This is a multidisciplinary, weekly meeting attended by spine surgeons, interventional radiologists, and radiation oncologists as well as the nurse practitioner and other members of the SPRO service, during which time cases are reviewed collaboratively and care plans are formulated in a coordinated fashion.

Furthermore, since its inception, the SPRO service has developed a more cohesive partnership with colleagues in palliative medicine. This not only includes interactions regarding patient care, but in 2013, weekly shared rounds were created to enhance our mutual learning and sharing of knowledge with respect to the many complexities of managing patients with advanced cancers.

**Program Growth**

Commensurate with the palliative therapy volume nationwide, within the DFBWCC Department of Radiation Oncology, approximately 40% of patients are receiving palliative RT. In the first year since SPRO’s establishment in 2011, the SPRO service saw approximately 850 consults (15.3 patients per week), in contrast to the approximate 10 consults per week in the year prior to the program’s inception. Over subsequent years (2012 to present), the service has experienced continued increase in volume, averaging approximately 16.2 consults per week. About 60% of patients are seen as inpatient consultations, and the remaining 40% are seen as outpatients.

**Aims of Supportive and Palliative Radiation Oncology**

The SPRO service aims to enhance the quality of palliative radiation oncology clinical care through *improving clinical operations* via the aforementioned dedicated, streamlined, and interdisciplinary service structure. This was illustrated in a 2013 survey-based study, executed to obtain feedback on SPRO’s impact on the quality of palliative cancer care and compare perceptions of palliative cancer care delivery among physicians practicing with and without a dedicated palliative radiation oncology service in the Boston area (Tseng et al., 2014).

The survey used a 7-point Likert scale with questions directed toward clinical caregivers’ perceptions of palliative cancer care within their organizations. A total of 117 care providers were contacted, and 102 responded (response rate of 89% for physicians, 83% for nurses), with no significant differences in characteristics between those who rated the SPRO-affiliated vs. nondedicated service departments.

Large majorities of radiation oncology care providers practicing within the DFBWCC believed that SPRO improved the quality of palliative cancer care, including te overall quality of care (physician/nurse, 98%/92%), communication with patients and families (95%/96%), staff experience (93%/84%), time spent on technical aspects of palliative cancer care (e.g., reviewing imaging; 88%/56%), appropriateness of treatment recommendations (85%/84%), appropriateness of dose/fractionation (78%/60%), and patient follow-up (64%/68%). Furthermore, in a comparison to physicians practicing in academic departments without a dedicated palliative radiation oncology service (who similarly rated measures of their departments’ palliative care quality), physicians at the SPRO-affiliated department rated the quality of their department’s palliative cancer care more highly (*p* = .02).

A second key aim of the SPRO service is to improve patient care through *optimized clinical communication* within radiation oncology along with other departments in our institutions. SPRO’s structure and daily workflow have been designed to enhance communication with other care teams, from the time of intake to the completion of consultations. This process includes having a single, call-in structure (pager), a dedicated administrator, and a dedicated care team with a systemized approach to communication with referring clinicians and other care providers.

SPRO has also streamlined and standardized communication within the radiation oncology department through a dedicated team with daily rounds. Furthermore, SPRO has optimized "handoffs" by minimizing their frequency (through a weekly rotating structure), creating a standard handoff structure as part of weekly rounds and employing an electronically based patient list containing critical sign-out information.

In addition, the roles of the nurse practitioners and SPRO administrator as nonrotating, dedicated service members also ensure the continuity of patient care from week to week. The SPRO team holds monthly meetings, which include SPRO staff and other departmental members integral to the care of SPRO patients (simulation therapists, lead treatment therapist, nursing, dosimetry, administrative support), with the goal of identifying issues and rendering effective changes to optimize patient care and clinical workflows in palliative oncology care.

A third aim of the SPRO service is *excellence in communication with patients and families*, who are typically facing urgent issues in the context of new or progressive advanced cancers. In light of this goal, SPRO team members have undergone additional training in advanced palliative care competencies, including communication with patients, families, and caregivers.

This training includes attending the Harvard Palliative Care Education and Practice course, which provides extensive instruction in advanced palliative medicine competencies, both in optimizing learners’ skills and their ability to educate others in those skills. The course includes intensive training in communication skills, such as conducting family meetings, interviewing patients, and facilitating end-of-life discussions. Furthermore, by being trained in how to teach others in advancing their communication skills, this course is aiding SPRO staff in developing as educators of other departmental staff and trainees in communication skills.

Finally, together with advancing clinical care in palliative radiation oncology, *education and research* to advance the quality of patient care are primary aims of the SPRO service. The SPRO service provides an ideal venue for dedicated training for departmental staff and trainees in palliative radiation oncology. Trainees frequently rotate on the service, which includes residents, fellows, nursing students, and medical students.

The SPRO service employs a variety of modalities for palliative oncology education, including clinical rounds discussions, interdisciplinary conferences (e.g., Palliative Care Teaching Rounds, Spine Oncology Conference), and dedicated lectures and case conferences on a variety of palliative oncology topics. Furthermore, the SPRO service is currently working on developing a formalized curriculum in palliative radiation oncology care. Additionally, the SPRO service, by centralizing palliative radiation oncology care, provides the structure necessary for research efforts to inform how to optimize the clinical care of patients and structures of care within palliative radiation oncology. To that end, a robust database has been constructed to store and access information, which can be utilized for research efforts.

**Relationship to the Needs of Other Departments**

In addition to the aforementioned goals, the SPRO service also aims to meet the needs of other departments in caring for advanced cancer patient—specifically medical oncology, surgical oncology, and palliative care—by creating a readily available and easily accessible central structure for palliative radiation oncology care. Moreover, the SPRO service is strongly aligned with the Dana-Farber Cancer Institute Department of Psychosocial Oncology and Palliative Care.

The Department of Psychosocial Oncology and Palliative Care provided direct input in the development of the SPRO service and regularly offers ongoing support and input. The SPRO service is also strongly aligned with the activities and goals of this Department, which are to "enhance quality of life and well-being and to relieve suffering in all its dimensions throughout illness, survivorship, and bereavement. The Department aims to contribute to the knowledge base guiding psychosocial and palliative interventions across the illness spectrum, train the next generation of leaders in psychosocial oncology and palliative care, and share expertise with students and colleagues in other disciplines" (Department of Psychosocial Oncology and Palliative Care, 2014).

**Future Directions**

Ongoing goals of the SPRO service are to further develop education and research to improve palliative radiation oncology clinical care. Future goals in educational efforts include the continued development of a palliative radiation oncology educational curriculum for residents and departmental staff. This project is currently underway and is being led by both radiation oncologists and nurse practitioners on the SPRO team. Core competencies in palliative care within radiation oncology have not yet been established, and therefore, as a part of curriculum development, we have been working with leaders in palliative radiation therapy and palliative medicine to determine what these core competencies should be.

Furthermore, continued advancement of research in palliative radiation oncology care is required, with goals including the development of a prospective database, which employs patient-reported outcomes, and partnering with other academic institutions in palliative radiation oncology research. Finally, the program aims to provide leadership in spearheading an academic emphasis on palliative care within radiation oncology.

## CONCLUSIONS

The Supportive and Palliative Radiation Oncology service aims to advance clinical care, education, and research in palliative radiation oncology care. Since its implementation in June 2011, it has demonstrated evidence of improvement in the quality of palliative radiation oncology patient care. It has likewise proved to be a centralized venue for education and research within palliative radiation oncology—key elements to advancing clinical care in this field. Much still needs to be done to promote palliative radiation oncology care, but the rapid uptake of the SPRO service at an academic center suggests such dedicated palliative radiation oncology systems of care are required to meet the needs of both patients and health-care systems.

## References

[1] Bernier Jacques, Hall Eric J, Giaccia Amato (2004). Radiation oncology: a century of achievements.. *Nature reviews. Cancer*.

[2] Chow E., Holden L. (2011). Rapid response radiotherapy program and bone metastasis site group clinic: Annual report. http://sunnybrook.ca/uploads/RRRPBMC_2011.pdf.

[3] Connell Philip P, Hellman Samuel (2009). Advances in radiotherapy and implications for the next century: a historical perspective.. *Cancer research*.

[4] Department of Psychosocial Oncology and Palliative Care. *2014*.

[5] Mitin T., Loeffler J. S., Ross M. E. (2013). Radiation therapy techniques in cancer care. http://www.uptodate.com/contents/radiation-therapy-techniques-in-cancer-treatment.

[6] Peppercorn Jeffrey M, Smith Thomas J, Helft Paul R, Debono David J, Berry Scott R, Wollins Dana S, Hayes Daniel M, Von Roenn Jamie H, Schnipper Lowell E (2011). American society of clinical oncology statement: toward individualized care for patients with advanced cancer.. *Journal of clinical oncology : official journal of the American Society of Clinical Oncology*.

[7] Tseng Y. D., Krishnan M. S., Jones J. A., Sullivan A. J., Gorman D., Taylor A., Balboni T. A. (2014). Supportive and palliative radiation oncology service: Impact of a dedicated service on palliative cancer care. *Practical Radiation Oncology*.

[8] van Oorschot Birgitt, Rades Dirk, Schulze Wolfgang, Beckmann Gabriele, Feyer Petra (2011). Palliative radiotherapy--new approaches.. *Seminars in oncology*.

